# GC-MS Analysis of Biological Nitrate and Nitrite Using Pentafluorobenzyl Bromide in Aqueous Acetone: A Dual Role of Carbonate/Bicarbonate as an Enhancer and Inhibitor of Derivatization

**DOI:** 10.3390/molecules26227003

**Published:** 2021-11-19

**Authors:** Dimitrios Tsikas

**Affiliations:** Core Unit Proteomics, Institute of Toxicology, Hannover Medical School, 30625 Hannover, Germany; Tsikas.dimitros@mh-hannover.de

**Keywords:** acetazolamide, carbonic anhydrase, derivatization, enhancement, GC-MS, inhibition, pentafluorobenzyl bromide

## Abstract

Carbon dioxide (CO_2_) and carbonates, which are widely distributed in nature, are constituents of inorganic and organic matter and are essential in vegetable and animal organisms. CO_2_ is the principal greenhouse gas in the atmosphere. In human blood, CO_2_/HCO_3_^−^ is an important buffering system. Inorganic nitrate (ONO_2_^−^) and nitrite (ONO^−^) are major metabolites and abundant reservoirs of nitric oxide (NO), an endogenous multifunctional signaling molecule. Carbonic anhydrase (CA) is involved in the reabsorption of nitrite and nitrate from the primary urine. The measurement of nitrate and nitrite in biological samples is of particular importance. The derivatization of nitrate and nitrite in biological samples alongside their ^15^N-labeled analogs, which serve as internal standards, is a prerequisite for their analysis by gas chromatography–mass spectrometry (GC-MS). A suitable derivatization reagent is pentafluorobenzyl bromide (PFB-Br). Nitrate and nitrite are converted in aqueous acetone to PFB-ONO_2_ and PFB-NO_2_, respectively. PFB-Br is also useful for the GC-MS analysis of carbonate/bicarbonate. This is of particular importance in conditions of pharmacological CA inhibition, for instance by acetazolamide, which is accompanied by elevated concomitant excretion of nitrate, nitrite and bicarbonate, as well as by urine alkalization. We performed a series of experiments with exogenous bicarbonate (NaHCO_3_) added to human urine samples (range, 0 to 100 mM), as well as with endogenous bicarbonate resulting from the inhibition of CA activity in healthy subjects before and after ingestion of pharmacological acetazolamide. Our results indicate that bicarbonate enhances the derivatization of nitrate with PFB-Br. In contrast, bicarbonate decreases the derivatization of nitrite with PFB-Br. Bicarbonate is not a catalyst, but it enhances PFB-ONO_2_ formation and inhibits PFB-NO_2_ formation in a concentration-dependent manner. The effects of bicarbonate are likely to result from its reaction with PFB-Br to generate PFB-OCOOH. Nitrate reacts with concomitantly produced PFB-OCOOH to form PFB-ONO_2_ in addition to the direct reaction of nitrate with PFB-Br. By contrast, nitrite does not react with PFB-OCOOH to form PFB-NO_2_. Sample acidification by small volumes of 20 wt.% aqueous acetic acid abolishes the effects of exogenous and endogenous bicarbonate on nitrite measurement.

## 1. Introduction

Pentafluorobenzyl bromide (2,3,4,5,6-pentafluorobenzyl bromide, PFB-Br) is a useful derivatization reagent for different classes of organic substances including carboxylic acids and amines, as well as inorganic anions, including nitrate and nitrite [1]. Derivatization with PFB-Br can be performed in water-free organic solvents such as acetonitrile, as well as in aqueous systems in the presence of water-miscible organic solvents such as acetone. Use of an acetone-aqueous sample in a volume ratio of 4:1 enables derivatization in homogenous phase [1]. Reactions with PFB-Br are nucleophilic substitutions of bromide by a nucleophile, which can be water, halogenides such as chloride, and other inorganic ions. The derivatization time is dependent upon the nucleophilicity and other factors, such as the stability of the PFB derivatives. The reaction products are lipophilic and extractable into organic solvents such as toluene and are best suitable for gas chromatography–mass spectrometry (GC-MS) analysis. Due to the fluorine atoms in PFB derivatives, their GC-MS analysis in the negative-ion chemical ionization mode revealed the highest sensitivity.

**Scheme 1:**
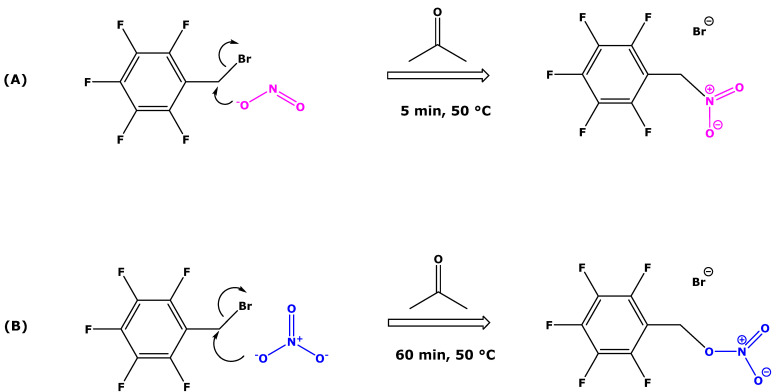
Derivatization of (**A**) nitrite and (**B**) nitrate in aqueous acetone with pentafluorobenzyl bromide at 50 °C. The optimum derivatization time is 5 min for nitrite and 60 min for nitrate.

We previously developed a GC-MS method for the simultaneous quantitative measurement of nitrite and nitrate in different biological samples including human plasma, urine and saliva [2]. The reaction of PFB-Br with nitrite in aqueous acetone leads to the formation of the nitro derivative (PFB-NO_2_), yet not of the expected nitrous acid ester (PFB-ONO) (Scheme 1, reaction A). The reaction of PFB-Br with nitrate generates the nitric acid ester (PFB-ONO_2_) (Scheme 1, reaction B). Kinetic investigations showed that nitrite reacts with PFB-Br more rapidly and to a higher extent than nitrate, even at room temperature [2]. Yet, PFB-NO_2_ seems to be readily susceptible to hydrolysis. As a compromise, we measured nitrite and nitrate simultaneously using GC-MS after derivatization with PFB-Br at 50 °C for 60 min. This procedure enhances the yield of the derivatization for nitrate and decreases the yield for nitrite due to hydrolysis. Yet, the use of the stable isotope analogs [^15^N]nitrite and [^15^N]nitrate guarantees highly accurate quantitative measurements in biological samples [2].

Carbon dioxide (CO_2_) and carbonates are widely distributed in nature. In human blood, CO_2_/HCO_3_^−^ is an important buffering system. In human urine, carbonate and bocrabonate are physiologically excreted [3,4]. Like nitrite and nitrate, we found that carbonate can react with PFB-Br under experimental conditions similar to those of nitrite and nitrate [5]. We observed the formation of the expected PFB-OCOOH derivative, albeit in low yield [5]. This behavior resembles in part that of nitrite derivatization with PFB-Br, which did not form any isolable PFB-ONO but exclusively (PFB-NO_2_).

In previous work, we observed an interaction of carbonate/bicarbonate with the analysis of nitrite in urine samples of subjects who took acetazolamide [6], a clinical drug [7]. Acetazolamide inhibits carboanhydrase (CA) activity in the kidneys, and because of this, it increases the excretion of bicarbonate and pH of the urine. Urine acidification of carbonate-containing urine samples with acetic acid (20 wt.%) did not influence the derivatization of nitrate but seemed to increase the yield of PFB-NO_2_ [6]. In the present work, we investigated the derivatization of nitrite and nitrate in human urine and plasma under various conditions, aiming to reveal potential mechanisms and solutions for the derivatization of nitrite and nitrate in the presence of high carbonate/bicarbonate concentrations.

## 2. Materials and Methods

### 2.1. Chemicals and Materials

2,3,4,5,6-Pentafluorobenzyl bromide (PFB-Br), sodium nitrite (purity 99.99+%), sodium [^15^N]nitrite and sodium [^15^N]nitrate (declared as 99 atom% at ^15^N each) were obtained from Sigma-Aldrich (Steinheim, Germany). Toluene was purchased from Baker (Deventer, The Netherlands). Sodium bicarbonate and carbonate, acetone and glacial acetic acid were from Merck (Darmstadt, Germany). ^2^H-Labelled creatinine ([*methylo*-^2^H_3_]creatinine, >99 atom% ^2^H) was obtained from Aldrich. PFB-Br is corrosive and an eye irritant. Inhalation and contact with skin and eyes should be avoided. All work should be and was performed in a well-ventilated fume hood. Separate stock solutions of salts were prepared by dissolving accurately weighed amounts of commercially available unlabeled and stable-isotope-labeled salts in deionized water. Stock solutions were diluted with deionized water as appropriate.

Glassware for GC-MS (1.5-mL autosampler vials and 0.2-mL microvials) including the fused-silica capillary column Optima 17 (15 m × 0.25 mm I.D., 0.25-micrometer film thickness) were purchased from Macherey-Nagel (Düren, Germany).

### 2.2. Derivatization Procedures for Nitrite and Nitrate and GC-MS Analyses

In general, the following derivatization procedure was used. Deviations are reported in the individual experiments. A total of 100-µL aliquots of a sample were added to 400-µL aliquots of acetone and 10-µL aliquots of PFB-Br, and the samples were heated at 50 °C for 5 min or 60 min. After derivatization, acetone was removed under a stream of nitrogen, and analytes were extracted by vortexing with toluene (1 mL). Nitrite and nitrate were measured simultaneously in 100 µL urine specimens by a previously reported, fully validated GC-MS method immediately after acidification by using a 20 wt.% acetic acid solution and derivatization by PFB-Br, as described elsewhere [2]. ^15^N-Labelled nitrite (final concentration, 4 µM or 1 µM) and ^15^N-labeled nitrate (final concentration, 400 µM or 100 µM) were used as internal standards for urinary nitrite and nitrate, respectively. To investigate the effect of the CO_2_/Na_2_CO_3_/NaHCO_3_ system on nitrite and nitrate analysis, samples were derivatized before and after acidification by 20 wt.% acetic acid to reach a final pH value of about 4.5 in order to remove CO_2_ from urine samples, as described elsewhere [2]. Urinary excretion of nitrite and nitrate was corrected for urinary creatinine excretion. Creatinine-corrected excretion rates are reported as µmol of nitrite or nitrate per mmol of creatinine.

Aliquots (1 µL) of the toluene extracts were injected into the GC-MS apparatus (model DSQ from ThermoFisher; Dreieich, Germany) in the splitless mode. Quantification was performed by selected-ion monitoring (SIM) of mass-to-charge (*m*/*z*) of *m*/*z* 46 for [^14^N]nitrite, *m*/*z* 47 for [^15^N]nitrite, *m*/*z* 62 for [^14^N]nitrate and *m*/*z* 63 for [^15^N]nitrate using a dwell time of 50 ms for each ion. The measured peak area (PA) values of unlabeled and labeled nitrite and nitrate and the peak area ratio (PAR) of unlabeled to labeled nitrite or nitrate were used in calculations.

### 2.3. Analyses in Urine Samples Collected in Previous Studies

In the present study, we used urine samples collected in a previously reported study [6], which had been performed as follows in brief. Six apparently healthy volunteers (2 females, aged 25 and 44 years; 4 males, aged 24–49 years) had participated in the study. In the morning (8 a.m.), the volunteers were orally given one to two tablets acetazolamide (Acemit^®^ 250 mg, medphano/Berlin, Germany) corresponding to a dose of about 5 mg/kg bodyweight. First, volunteers emptied their bladder and collected the first urine specimen (time—2 h) followed by two collections at time—1 h and time 0 h. Immediately after collection of the 0 h urine sample, acetazolamide was taken by a glass of drinking water. Four urine samples were collected in polypropylene tubes in 30 min intervals and another four urine samples in 60 min intervals subsequently. Immediately after each collection, tubes were closed and put on ice. Urine samples were collected at several time points before and after acetazolamide ingestion, portioned in 1 mL and 10 mL aliquots and stored either at +5 °C for pH and carbonate measurement on the same day, or at −20 °C until analysis for nitrite, nitrate and creatinine on next day.

In some analyses, spot urine was collected by the author without any medication and used in some in the vitro studies on the effects of bicarbonate on nitrite and nitrate analyses.

### 2.4. Statistical Analysis

Results are expressed as mean with standard error of the mean, or as mean with standard deviation, as specified in the respective experiments. Differences between neighbor values were analyzed with paired or unpaired *t* tests as appropriate. *p* values ≤ 0.05 were considered statistically significantly different. Calculations and graphs were performed using GraphPad Prism version 7.0 (San Diego, CA, USA).

## 3. Results

### 3.1. Effect of Exogenous Bicarbonate and Hydroxide on the Derivatization of [^15^N]nitrate, [^15^N]nitrite and Endogenous Nitrate and Nitrite in Human Urine

**Figure 1:**
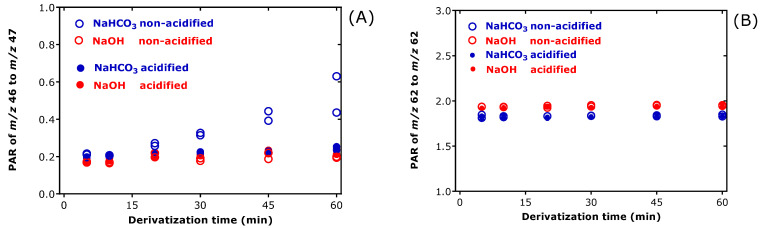
Effects of exogenous NaHCO_3_ and NaOH on nitrite and nitrate analysis by GC-MS after derivatization with PFB-Br for the indicated times at 50 °C in a pooled human urine sample treated with 100 mM NaHCO_3_ or with 100 mM NaOH. The samples were derivatized before and after acidification with 20 wt.% acetic acid. The concentrations of the internal standards in the urine samples were 400 µM [^15^N]nitrate and 4 µM [^15^N]nitrite. Data are shown from two independent experiments. All nitrite and nitrate species were measured simultaneously by GC-MS using selected-ion monitoring of (**A**) *m*/*z* 46 for [^14^N]nitrite and *m*/*z* 47 for [^15^N]nitrite, and of (**B**) *m*/*z* 62 for [^14^N]nitrate and *m*/*z* 63 for [^15^N]nitrate.

A healthy volunteer provided a morning urine. The freshly collected urine sample was spiked with 400 µM [^15^N]nitrate and 4 µM [^15^N]nitrite. This pooled urine sample was divided into two equal fractions that were spiked with freshly prepared 100 mM NaHCO_3_ or 100 mM NaOH. Thereafter, each two 100 µL aliquots of the urine samples were derivatized with PFB-Br at 50 °C for varying times (0, 5, 10, 20, 30, 45 and 60 min) without prior acidification, as well as after acidification with 20 wt.% acetic acid. All nitrite and nitrate species were measured simultaneously by GC-MS using SIM of *m*/*z* 46 for [^14^N]nitrite, *m*/*z* 47 for [^15^N]nitrite, *m*/*z* 62 for [^14^N]nitrate and *m*/*z* 63 for [^15^N]nitrate. The main results of this experiment are shown in Figure 1.

The PAR of *m*/*z* 46 to *m*/*z* 47 for nitrite and the PAR of *m*/*z* 62 to *m*/*z* 63 for nitrate behaved differently in the two urine samples. On the other hand, the PAR values behaved very similarly in the acidified urine samples that originally contained 100 mM NaHCO_3_ or 100 mM NaOH. The greatest differences between the NaHCO_3_- and NaOH-treated urine samples were observed for nitrite. The PAR of *m*/*z* 46 to *m*/*z* 47 increased after 20 min of derivatization only in the non-acidified, NaHCO_3_-treated urine sample. This finding strongly indicates that it is not the alkalinity, but the CO_2_/Na_2_CO_3_/NaHCO_3_ system of the urine that interferes with the analysis of nitrite in human urine. This interference can be eliminated by acidification of the urine sample, most likely by instantaneous conversion of Na_2_CO_3_ and NaHCO_3_ to highly volatile CO_2_ [8].

### 3.2. Effect of Exogenous Bicarbonate on the Derivatization of [^15^N]nitrate, [^15^N]nitrite and Endogenous Nitrate and Nitrite in Human Urine

**Figure 2:**
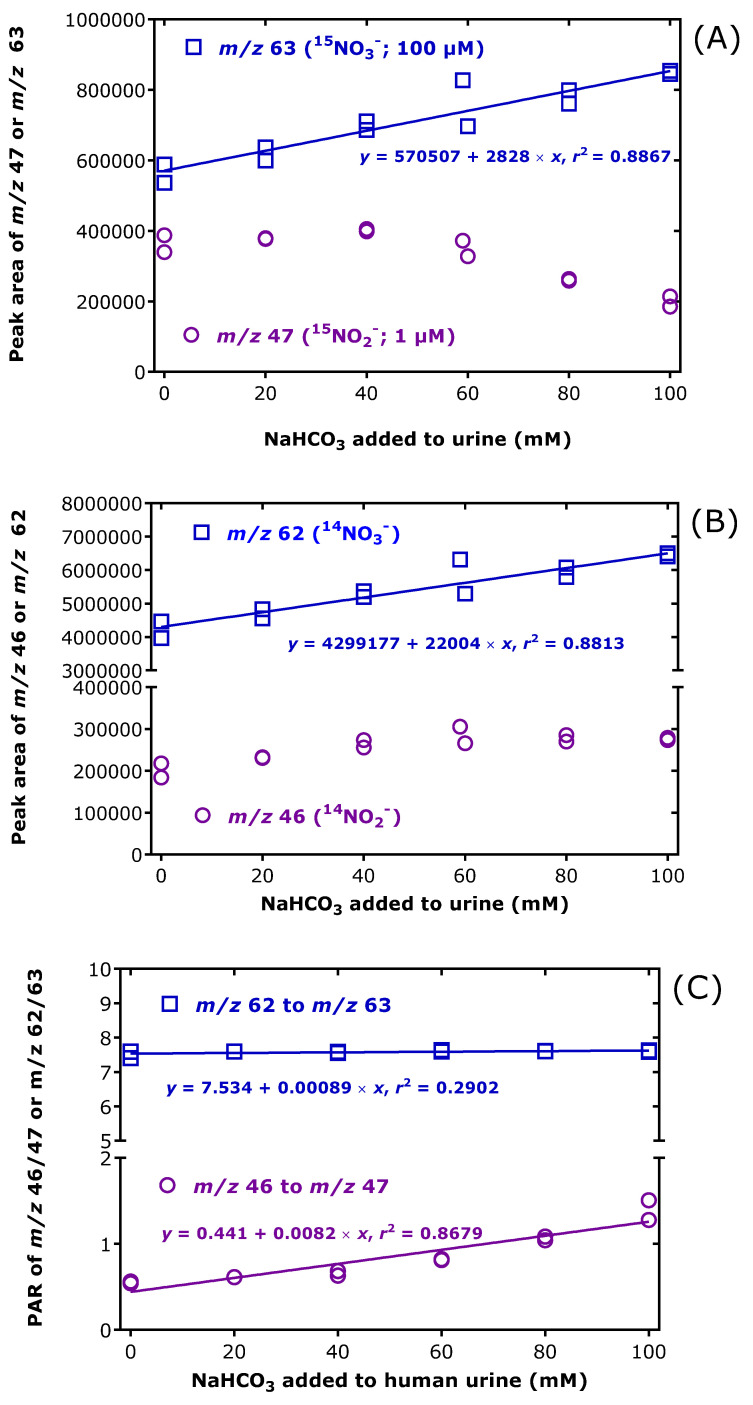
Plots of the peak area of (**A**) *m*/*z* 63 for [^15^N]nitrate and *m*/*z* 47 for [^15^N]nitrite, of (**B**) *m*/*z* 62 for [^14^N]nitrate and *m*/*z* 46 for [^14^N]nitrite in a human urine sample spiked with the indicated bicarbonate concentrations, and of (**C**) the peak area ratio (PAR) of *m*/*z* 62 to *m*/*z* 63 for nitrate and of *m*/*z* 46 to *m*/*z* 47 for nitrite. The added concentrations were 100 µM for [^15^N]nitrate and 1 µM for [^15^N]nitrite. Data are shown from two independent experiments. The derivatization time with PFB-Br was each 60 min.

A pooled human urine sample was spiked with [^15^N]nitrate and [^15^N]nitrite at final concentrations of 100 µM and 1 µM, respectively. Each two 100 µL aliquots of this sample were spiked with an aqueous solution of NaHCO_3_ to reach final added concentrations of 0, 20, 40, 60, 80 and 100 mM. After immediate derivatization with PFB-Br for 60 min, the derivatives were extracted with toluene (1 mL) and 1 µL aliquots thereof were analyzed by GC-MS in the SIM mode. Figure 2A shows that the peak area of [^15^N]nitrate increases linearly with increasing concentration of added NaHCO_3_, whereas the peak area of [^15^N]nitrite decreases at added NaHCO_3_ concentrations of 60, 80 and 100 mM. Figure 2B shows that the peak area of endogenous nitrate (i.e., [^14^N]nitrate) increases linearly with increasing concentration of added NaHCO_3_, whereas the peak area of endogenous nitrite (i.e., [^14^N]nitrite) increases at added NaHCO_3_ concentrations up to 60 mM with a tendency to slightly decrease at 80 mM and 100 mM NaHCO_3_. Figure 2C shows the PAR of *m*/*z* 46 to *m*/*z* 47 for nitrite and the PAR of *m*/*z* 62 to *m*/*z* 63 for nitrate. The PAR of *m*/*z* 62 to *m*/*z* 63 is constant, i.e., independent of the added NaHCO_3_ concentration. The mean PAR *m*/*z* 62 to *m*/*z* 63 was 7.579 (RSD, 0.8%) corresponding to a concentration of 758 µM for endogenous nitrate in the urine sample. Previously, we found that the derivatization of nitrate with PFB-Br is incomplete even for 60 min at 50 °C [2]. The results of Figure 2 suggest that the formation of the PFB-O^15^NO_2_ and PFB-O^14^NO_2_ increases to the same extent in dependency on the NaHCO_3_ concentration in the urine. Thus, nitrate can be reliably measured in human urine in the presence of high bicarbonate concentrations, as they may occur upon acetazolamide administration [6]. On the other hand, the results of Figure 2 indicate that NaHCO_3_, at concentration of 60, 80 and 100 mM, decreases the formation of PFB-^15^NO_2_ and PFB-^14^NO_2_. This may eventually lead to overestimation and thus to inaccurate measurement of nitrite in urine samples that contain high concentrations of bicarbonate.

### 3.3. Effects of Exogenous and Endogenous Bicarbonate, Acidification and Derivatization Time on the Derivatization of Nitrate and Nitrite in Human Urine

**Figure 3:**
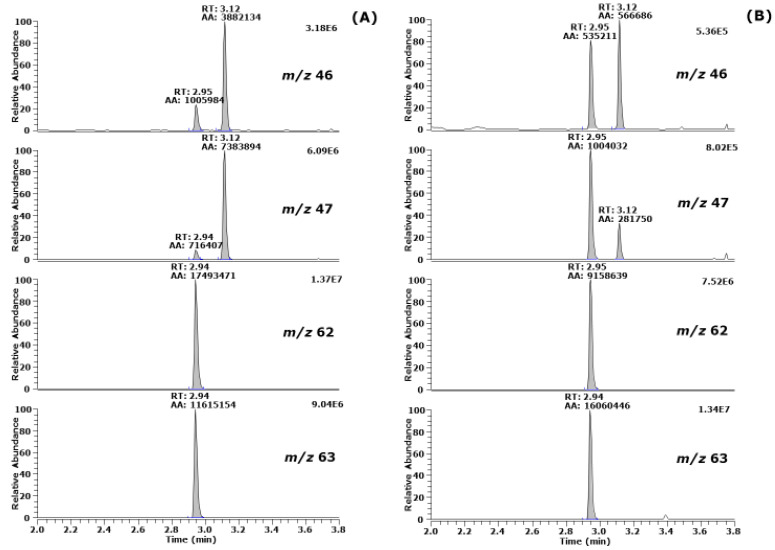
Representative partial GC-MS chromatograms from the simultaneous analysis of nitrite and nitrate in human urine samples without external addition of NaHCO_3_ (**A**) and with addition of 100 mM NaHCO_3_ (**B**). Derivatization with PFB-Br for 60 min at 50 °C in aqueous acetone was performed. The concentrations of the internal standards in the urine samples were 400 µM [^15^N]nitrate and 4 µM [^15^N]nitrite. GC-MS analysis was performed using selected-ion monitoring of *m*/*z* 46 for [^14^N]nitrite, *m*/*z* 47 for [^15^N]nitrite, *m*/*z* 62 for [^14^N]nitrate and *m*/*z* 63 for [^15^N]nitrate. The retention time (RT) was 2.95 min for nitrate and 3.12 min for nitrite. Note the decrease of the intensity and the peak area values of *m*/*z* 46 for [^14^N]nitrite and *m*/*z* 47 for [^15^N]nitrite in presence of bicarbonate (**B**). AA means peak area calculated in the automated mode. The ThermoFisher quadrupole GC-MS apparatus model DSQ in the negative-ion chemical ionization mode was used.

**Figure 4:**
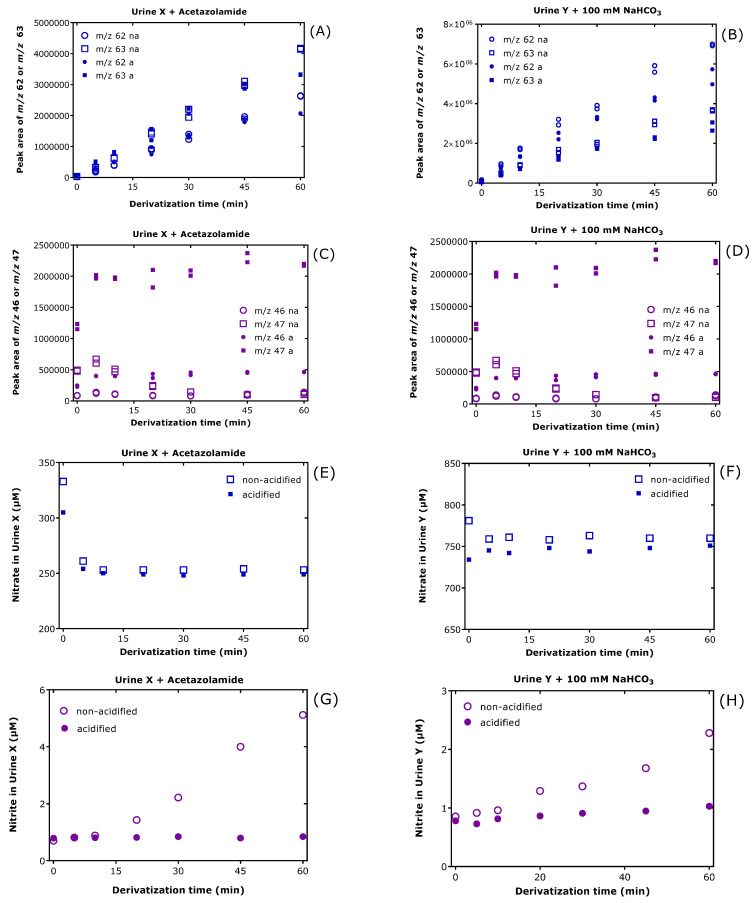
Effects of exogenous bicarbonate and of the ingestion of acetazolamide on nitrate and nitrite analysis by GC-MS after derivatization with PFB-Br for the indicated times at 50 °C in two different human urine samples. The samples were derivatized before and after acidification with 20 wt.% acetic acid. The concentrations of the internal standards in the urine samples were 400 µM [^15^N]nitrate and 4 µM [^15^N]nitrite. Urine X (**A**,**C**,**E**,**G**) was collected upon ingestion of a 500-milligram tablet acetazolamide from a previous study [6]. Urine Y (**B**,**D**,**F**,**H**) was freshly collected by a healthy volunteer (author of the present article) and spiked with 100 mM NaHCO_3_. Data are shown from two independent experiments. All nitrite and nitrate species were measured simultaneously by GC-MS using selected-ion monitoring of *m*/*z* 46 for [^14^N]nitrite, *m*/*z* 47 for [^15^N]nitrite, (**B**) *m*/*z* 62 for [^14^N]nitrate and *m*/*z* 63 for [^15^N]nitrate. Left panels, Urine X; right panels, Urine Y. Acidified, close symbols; non-acidified, open symbols.

Two pooled human urine samples, i.e., Urine X and Urine Y, were spiked with [^15^N]nitrate and [^15^N]nitrite at the final added concentrations of 400 µM and 4 µM, respectively. Urine X was obtained from a volunteer who orally received acetazolamide. Urine Y was collected by another volunteer who did not receive any drug. The concentrations of bicarbonate, nitrate and nitrite were unknown in both urine samples. Urine Y was freshly spiked with 100 mM NaHCO_3_. Aliquots (100 µL) of the urines were derivatized with PFB-Br at 50 °C for different incubation times (range, 0 to 60 min) without and with acidification of the samples and analyzed by GC-MS as described above. The GC-MS chromatograms from these analyses are shown in Figure 3. The results of this experiment are illustrated in Figure 4.

The effects of the derivatization time and acidification of the urine samples were qualitatively closely comparable. The peak area values of *m*/*z* 62 and *m*/*z* 63 increased with derivatization time and were lower in the acidified samples of Urine X and Urine Y (Figure 4A,B). The peak area values of *m*/*z* 46 and *m*/*z* 47 changed with derivatization time and were higher in the acidified samples of Urine X and Urine Y (Figure 4C,D). The highest peak area values were obtained at the derivatization time of 5 min. With the exception of the derivatization time of 0 min (urine sample was treated with PFB-Br and extracted immediately), the concentration of endogenous nitrate was independent of the derivatization time, but it was constantly lower in the acidified urine samples (Figure 4E,F). The concentration of endogenous nitrite was independent of the derivatization time of the acidified samples, but it increased constantly with the derivatization time larger than 10 min (Figure 4G,H).

### 3.4. Effect of Exogenous Bicarbonate on the Derivatization of [^15^N]nitrite and Endogenous Nitrite in Human Plasma

**Figure 5:**
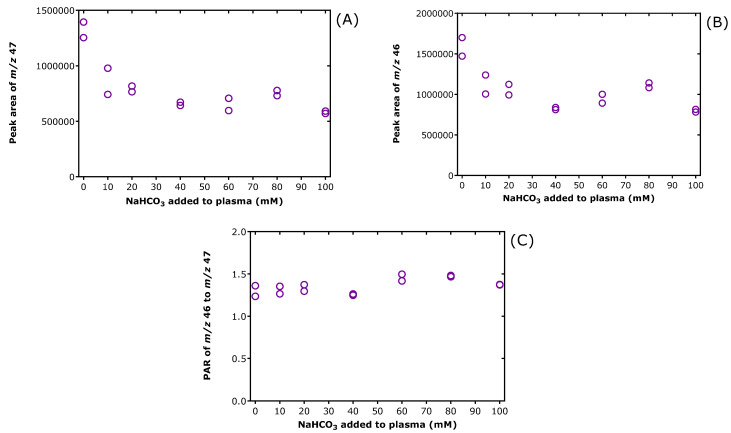
Plots of the peak area of (**A**) *m*/*z* 47 for exogenous nitrite (i.e., [^15^N]nitrite, 1 µM), of (**B**) *m*/*z* 46 for endogenous nitrite (i.e., [^14^N]nitrite) in a human plasma sample spiked with the indicated bicarbonate concentrations, and of (**C**) the resulting peak area ratio (PAR) of *m*/*z* 46 to *m*/*z* 47 for nitrite. Data are shown from two independent experiments. The derivatization time with PFB-Br was 5 min each. No [^15^N]nitrate was added to the plasma sample.

A pooled human plasma sample was spiked with [^15^N]nitrite at a final concentration of 1 µM. Each two 100-µL aliquots of this sample were spiked with an aqueous solution of NaHCO_3_ to reach final added concentrations of 0, 10, 20, 40, 60, 80 and 100 mM. After derivatization with PFB-Br for 5 min, the derivatives were extracted with toluene and analyzed by GC-MS in the SIM mode. The results of these measurements are shown in Figure 5.

The peak area of *m*/*z* 47 for the internal standard [^15^N]nitrite and of *m*/*z* 46 for the endogenous nitrite ([^14^N]nitrite) decreased considerably at added NaHCO_3_ concentrations of 10 mM, 20 mM and 40 mM (Figure 5A,B). However, the PAR of *m*/*z* 46 to *m*/*z* 47 was largely independent of the NaHCO_3_ concentration (Figure 5C). The mean PAR was 1.357 (6.43%), indicating a concentration of 1.36 µM for endogenous nitrite in the plasma sample. These results suggest that the exogenous NaHCO_3_ concentration equally inhibits the formation of PFB-^15^NO_2_ and PFB-^14^NO_2_ by maximally 50% under these conditions. Thus, the endogenous CO_2_/Na_2_CO_3_/NaHCO_3_ system, which amounts to about 20 mM in total of human blood, is likely to inhibit the derivatization of nitrite with PFB-Br, yet without affecting analytical reliability in terms of accuracy. Unlike urinary nitrite, sample acidification is not pressingly needed in the quantitative analysis of nitrite in human plasma using PFB-Br derivatization.

## 4. Discussion

The derivatization of organic and inorganic anions such as nitrite, nitrate, chloride and carbonate is an indispensable analytical procedure in gas chromatography-based techniques for the vast majority of natural compounds. Pentafluorobenzyl bromide (PFB-Br) is a highly versatile derivatization reagent because of its favorable physicochemical properties with respect to both chromatography and detection due to its strongly electron-capturing F atoms [1]. The latter property leads to unbeatable amol-sensitivity in GC-MS-based approaches operating in the chemical ionization mode for numerous analytes in virtually all kinds of biological samples. As endogenous metabolites of NO and of environmental NO_x_ species produced by human and natural activities, nitrite and nitrate are of general interest. Many different methods have been reported for the analysis of nitrite and nitrate in the last two centuries [9]. We found that endogenous nitrite and nitrate can be simultaneously derivatized with PFB-Br in numerous biological fluids and tissues in their acetonic solutions and suspensions [2]. Nitrite and nitrate are converted by PFB-Br to PFB-NO_2_ and PFB-ONO_2_, respectively, virtually without the need of any catalyst. Nitrite and nitrate can be simultaneously quantitated by GC-MS as PFB-NO_2_ and PFB-ONO_2_ by using commercially available salts of [^15^N]nitrite and [^15^N]nitrate as internal standards, without problems arising from the need to chemically or enzymatically reduce nitrate to nitrite prior to derivatization.

**Figure 6:**
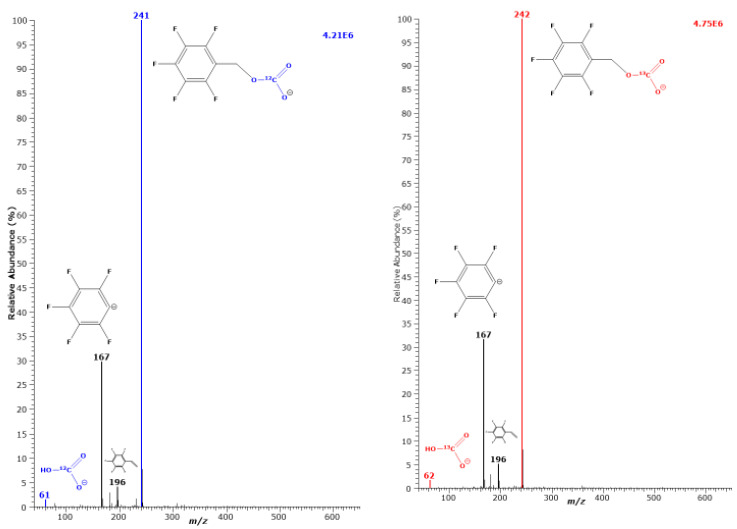
Negative-ion chemical ionization GC-MS spectra of PFB-O^12^COOH and PFB-O^13^COOH, two minor derivatization products from the separate reaction of ^12^C- and ^13^C-carbonate in aqueous acetone with pentafluorobenzyl bromide (PFB-Br) for 60 min at 50 °C. PFB-O^12^COOH (M, 242.1) and PFB-O^13^COOH (M, 243.1) have the same retention time of 6.97 min. Inserts indicate the structure of the proposed anions of ^12^C-carbonate (left, blue) and ^13^C-carbonate (right, red). The anions *m*/*z* 167 and *m*/*z* 196 are derived from the derivatization reagent PFB-Br. These mass spectra have been previously reported as supplementary information in Ref. [5].

**Scheme 2:**
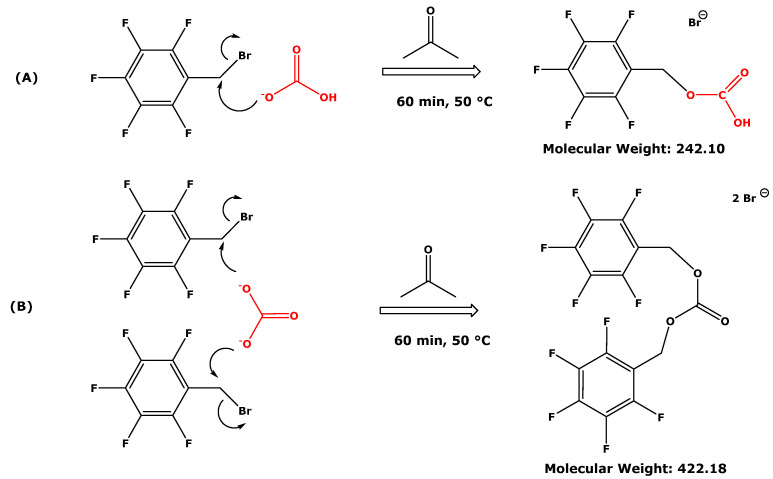
Minor derivatization products from the reaction of carbonate in aqueous acetone with (**A**) one and (**B**) two pentafluorobenzyl bromide molecules at 50 °C. PFB-OCOOH (M, 241.1); PFB-O(CO)-O-PFB (M. 422.2).

PFB-Br is also suitable for the analysis of carboxylic groups-containing compounds in water-free organic solvents. Yet, the derivatization of fatty acids and their metabolites with PFB-Br requires the use of an organic base as a catalyst. Captopril is a carboxylic drug, and its derivatization with PFB-Br to its PFB ester has been reported to be catalyzed by carbonate [10]. Carbonate is often used in analytical derivatization, for instance, that of dimethylamine with pentafluorobenzoyl chloride, notably by means of extractive pentafluorobenzoylation [11,12]. However, the underlying mechanism of the catalytic action of carbonate is not yet fully understood. Previously, we had no indication that the derivatization of nitrite and nitrate with PFB-Br required carbonate/bicarbonate as a catalyst. We found that PFB-Br is suitable for the derivatization of carbonate/bicarbonate under the derivatization conditions of nitrite and nitrate [5]. In aqueous acetone, carbonate was found to form many reaction products. Two major carbonate derivatives were identified as CH_3_COCH_2_−C(OH)(OPFB)_2_ and CH_3_COCH═C(OPFB)_2_, suggesting unique acetone-involving reactions. Two minor carbonate derivatives were PFB-OCOOH and O═CO_2_−(PFB)_2_. The GC-MS spectra of the PFB-O^12^COOH and PFB-O^13^COOH derivatives are shown in Figure 6 [5]. To the best of our knowledge, benzyl and pentafluorobenzyl esters of carbonate/bicarbonate have not been reported elsewhere, nor are they commercially available. PFB-OCOOH is presumably labile in aqueous solutions, but isolable by solvent extraction with toluene and apparently stable therein for GC-MS analysis [5]. It is proposed that PFB-OCOOH and O═CO_2_−(PFB)_2_ are formed by the reaction of carbonate with one and two PFB-Br molecules, respectively, yet without the incorporation of acetone in these derivatives (Scheme 2).

In experiments with acetazolamide, a diuretic drug that massively enhances the excretion of carbonate/bicarbonate in the urine, we observed that the derivatization of nitrite with PFB-Br in urine samples of humans who ingested acetazolamide was decreased, suggesting a strong inhibitory effect of the conversion of nitrite into PFB-NO_2_ [6]. This effect was abolished by acidifying the urine samples with aqueous acetic acid to pH values around 4.5 [6]. It is known that the derivatization of nitrite and halides with PFB-Br in its solutions in acetone, acetonitrile or ethanol are dependent on the pH value of the derivatization mixture [13]. It is also known that nitrite can react with CO_2_ [14]. Thus, the CO_2_/Na_2_CO_3_/NaHCO_3_ system can interfere with the PFB-Br derivatization of nitrite and nitrate in biological samples by several different mechanisms. In the present work, we addressed such potential mechanisms.

In order to gain more mechanistic information in this study, we used [^15^N]nitrite and [^15^N]nitrate, human urine and plasma samples spiked with NaHCO_3_ in relevant physiological and pharmacological concentration ranges. We also used urine samples from volunteers who took acetazolamide at pharmacological doses (around 5 mg/kg bodyweight).

The results of the present and previous studies suggest that carbonate does not catalyze the derivatization of nitrate to PFB-ONO_2_ with PFB-Br in homogenous phase in aqueous acetone. Carbonate/bicarbonate-containing biological samples, notably human urine, are by nature alkaline. However, it is not the alkalinity itself but the presence of carbonate/bicarbonate at high concentrations that is responsible in part for the massive impairment of nitrite derivatization with PFB-Br. The concentration of carbonate/bicarbonate in human plasma is of the order of 20 mM, that of nitrate in the range of 20 to 100 µM and that of nitrite of the order of 1 µM. The concentration of carbonate/bicarbonate in human urine is usually below 10 mM, that of nitrate of the order of 1000 µM and that of nitrite of the order of 10 µM. In the case of acetazolamide ingestion, blood carbonate/bicarbonate and blood pH only slightly change. However, in urine, carbonate/bicarbonate can reach concentrations up to about 100 mM upon acetazolamide intake, whereas nitrate and nitrite concentrations in urine instead decrease due to the diuretic effects of the drug. Thus, in such urine samples, carbonate/bicarbonate are present in a high molar excess of nitrate and especially of nitrite. As an example, we considered a urine sample that contains 10 mM carbonate/bicarbonate, 1000 µM nitrate and 10 µM nitrite. Under regular derivatization conditions (100 µL urine, 10 µL PFB-Br equivalent to 70 µmol), PFB-Br is present in a very high molar excess in carbonate/bicarbonate (69:1 µmol), nitrate (690:1) and nitrite (6900:1). In addition, human urine contains many other organic and inorganic substances at mM concentrations, such as creatinine [15] and chloride [16], which can react with PFB-Br under the same derivatization conditions. Thus, nitrate and nitrite compete with many other species for PFB-Br. High increases of the concentration of competing nucleophiles with small changes of nitrate and nitrite concentrations, for instance carbonate/bicarbonate, would decrease the molar ratio of PFB-Br to nitrate and nitrite. As the yield of derivatization reactions with PFB-Br also depends upon the PFB-Br concentration [13], high increases of competitive analytes would decrease the derivatization yield of PFB-NO_2_ and PFB-ONO_2_. Our studies show that increasing carbonate/bicarbonate concentrations decrease the yield of PFB-NO_2_, but they increase the yield of PFB-ONO_2_. One may therefore conclude that competition alone cannot explain the opposite effects of carbonate/bicarbonate on nitrate and nitrite derivatization with PFB-Br.

**Scheme 3:**
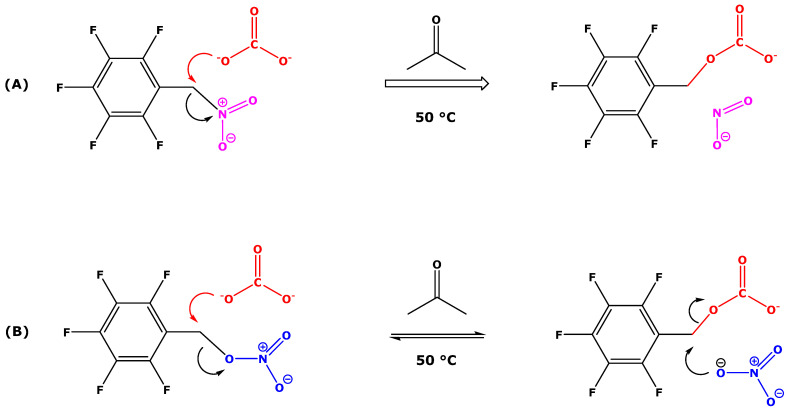
Proposed reactions for the nucleophilic substitution by carbonate from (**A**) PFB-NO_2_ and (**B**) PFB-ONO_2_ to release nitrite and nitrate, respectively, during the derivatization in aqueous acetone with pentafluorobenzyl bromide at 50 °C in the presence of carbonate. Reaction (**A**) is considered irreversible, reaction B is assumed reversible.

A more convincing assumption could be that intermediate derivatives of carbonate/bicarbonate with PFB-Br, notably PFB-OCOOH and PFB-O(CO)-O-PFB, interact with nitrate and nitrite to produce diametrically opposed effects on the conversion of nitrate to PFB-ONO_2_ and of nitrite to PFB-NO_2_ (Scheme 3). The nucleophilic attack of nitrate on the benzyl groups of PFB-OCOOH and PFB-O(CO)-O-PFB would then increase the formation of PFB-ONO_2_, thereby releasing carbonate. As PFB-ONO has not been detected thus far, it is possible that nitrite attacks the benzyl group of PFB-Br exclusively with its N atom to produce to PFB-NO_2_. This reaction occurs more rapidly and abundantly than the reaction of nitrate with PFB-Br to generate PFB-ONO_2_ [2]. That carbonate/bicarbonate inhibited the formation of PFB-NO_2_ suggests that nitrite cannot react with PFB-OCOOH and PFB-O(CO)-O-PFB to form PFB-NO_2_ and to release carbonate. Yet, it is also possible that carbonate reacts with PFB-NO_2_ and PFB-ONO_2_ to generate PFB-OCOOH, thereby releasing nitrite and nitrate, respectively (Scheme 3). Thus, the reaction of carbonate/bicarbonate with PFB-NO_2_ is considered irreversible and decreases the concentration of PFB-NO_2_ during the derivatization. On the other hand, the reaction of carbonate/bicarbonate with PFB-ONO_2_ is considered reversible and eventually increases the concentration of PFB-ONO_2_ during the derivatization, in addition to the direct reaction of nitrate with PFB-Br.

In theory, carbonate/bicarbonate may contribute to nitrate in the case of chromatographic co-elution of PFB-OCOOH and PFB-ONO_2_. This is because the ^13^C isotope of carbonate forms PFB-O^13^COOH, which ionizes to form *m*/*z* 62, albeit to a very low extent of about 2% (Figure 6). Such a contribution is considered very low because of the natural abundance of ^13^C of 1.1%. Nevertheless, the contribution of carbonate/bicarbonate to nitrate may be higher in the case that ^13^C-carbonate is used as the internal standard for endogenous carbonate/bicarbonate.

## 5. Conclusions

The derivatization of inorganic anions such as nitrite, nitrate and carbonate/bicarbonate in biological and environmental samples with PFB-Br using acetone as the organic solvent is performed in the homogenous phase and makes their GC-MS analysis possible. Although quantitative analysis is realized by using stable isotope labeled analogs, such as ^15^N-nitrite, ^15^N-nitrate and ^13^C-carbonate, interferences may occur due to their ubiquitous occurrence in the form of their contaminants. Another potential source of interference with the analysis of nitrite and nitrate in human urine may be the occurrence of carbonate/bicarbonate at manifold higher concentrations than under normal conditions. We identified such a condition when humans ingested pharmacological doses of the diuretic acetazolamide, an inhibitor of human CA II and CA IV. Acetazolamide potently inhibits renal CA activity and, in this way, massively increases the excretion of carbonate/bicarbonate in the urine and alkalizes the urine to pH values of about 8. Our studies indicate that carbonate/bicarbonate exerts diametrically opposed effects on the analysis of nitrite and nitrate by GC-MS when derivatized with PFB-Br. Carbonate/bicarbonate increases the formation of PFB-ONO_2_ but decreases the formation of PFB-NO_2_. These effects are not due to the concurrent alkalization of the urine by drugs such as acetazolamide. Rather, carbonate/bicarbonate increases the formation of PFB-ONO_2_ by enhancing the reaction of nitrate with an intermediate and isolable reaction product of carbonate/bicarbonate with PFB-Br, i.e., PFB-OCOOH. Unlike in other derivatization reactions such as with pentafluorobenzoyl chloride, carbonate/bicarbonate does not act as a catalyst in the derivatization of nitrate and nitrite with PFB-Br. On the other hand, carbonate/bicarbonate decreases the formation of PFB-NO_2_ most likely due the inability of nitrite to attack the PFB-OCOOH via its N atom. Such effects are much less pronounced in human plasma, in part because of the lower carbonate/bicarbonate concentration and in part due to the higher buffer capacity of the plasma compared to urine. A very simple and effective solution of the negative effect of high carbonate/bicarbonate concentrations on nitrite measurement in urine as PFB-NO_2_ is mild acidification by adding small volumes of 20 wt.% acetic acid to the urine.

## Data Availability

Not applicable.

## Sample Availability

Samples of the compounds are not available from the authors.
